# Evidence in All Policies: Restoring Science to Public Health and Clinical Decision-Making

**DOI:** 10.1007/s11606-026-10534-z

**Published:** 2026-05-26

**Authors:** Arnaud Chiolero, Nicolas Rodondi, Valérie Santschi

**Affiliations:** 1 Population Health Laboratory (#PopHealthLab), University of Fribourg, Fribourg, Switzerland; 2 School of Population and Global Health, McGill University, Montreal, QC Canada; 3 Institute for Primary Health Care (BIHAM), University of Bern, Bern, Switzerland; 4 Department of General Internal Medicine, Inselspital, Bern University Hospital, University of Bern, Bern, Switzerland; 5 La Source, School of Nursing Sciences, HES-SO University of Applied Sciences and Arts Western Switzerland, Lausanne, Switzerland

Public health and healthcare evidence are under attack in several countries. Scientific findings, produced at a massive scale and more accessible than ever, are too often ignored or distorted; misinformation spreads faster through the rise of social media and the digital transformation of information landscapes—a trend that has intensified in recent years.^1,2^ Inspired by the *Health in All Policies* approach—which seeks to systematically incorporate a health perspective into policymaking across all sectors—and its key component health impact assessment,^3^ we should now advocate for *Evidence in All Policies* to seek the systematic integration of scientific evidence into all aspects of public health and clinical decision-making.

In principle, *Evidence in All Policies* is neither novel nor complex. It rests on the straightforward idea that every public health and clinical decision should be informed by peer-reviewed information gathered from population health monitoring, evidence synthesis based on high-quality clinical studies, and the continuous evaluation of policies and clinical recommendations, and related population-level and clinical interventions.^3,4^ To provide this evidence-based information, we need autonomous and credible scientific institutions with staff trained in clinical and population health sciences, as well as in data-sciences and evidence synthesis.

This evidence-based policymaking process is well established in theory (Table 1).^3,4^ While never enough implemented, and challenged by the growing complexity of healthcare and public health knowledge and practice, it is highly worrisome to see that this process might recede as revealed recently in the USA, e.g., by the weakening in the evidence-based approach of scientific institutions like the Centers of Disease Control (CDC), the attempt to change the composition of the US Preventive Services Task Force (USPSTF), or the sacking of a vaccine committee that issues evidence-based recommendations on immunizations. Attacking the credibility of scientific institutions has also been observed in other countries especially related to vaccinations and health environmental issues.^5^ As a result, the scientific credibility of their observations or recommendations is jeopardized, and public health officials are no longer trusted.

**Table 1:** Key Characteristics of Evidence-Based (or Evidence-Informed) Public Health^5^

Characteristics
Making decisions using the best available peer-reviewed evidence
Using data and information systems systematically
Applying program-planning framework
Engaging the community in assessment and decision-making
Conducting sound evaluation
Disseminating what is learned to key stakeholders and decision-makers

How can we overcome these trends?

The barriers against the implementation of *Evidence in All Policies* are political and institutional more than technical. The critical problem is who produces the evidence because expertise, independence, and transparency in evidence generation are essential for science to inform health policy and clinical guidelines.^2^ However, many governments remain reluctant to cede control, preferring instead to manage directly the production and diffusion of information needed for decision-making.^6^ But when evidence is subordinate to politics (as well as to vested interest), integrity is in danger and trust disappears. Governments might select the production of evidence that justifies their policies or public health decisions,^6^ demonstrating how political forces can undermine science-based decision-making.

Further, evidence-informed decision-making is worsened by the infodemic (a term coined by the WHO to describe the overabundance of information disseminated in real time via multiple channels)^1^ and escalating spread of misinformation, the limited scientific and health literacy among both the public and policymakers, and the difficulty many health scientists face in communicating facts and uncertainties effectively beyond academia.^7^ The result is a widening gap between knowledge and public health and clinical practice—a space where intuition, common sense, ideology, and politics replace evidence.

To bridge this gap, countries must invest in—or protect from dismantlement—independent scientific institutions with clear mandates to produce and disseminate public health and clinical evidence, monitor the health of the population, provide risk assessment, and evaluate public health and healthcare practices. What is normal should be the possibility for these institutions to produce—safely—evidence at odds with government (or public) expectations or values. The dialogue between evidence producers and decision-makers is complex, sometimes conflictive, and needs to be organized because data and evidence do not speak for themselves—they must be interpreted and negotiated for their use and adoption. What is not normal, however, is the difficulty for these institutions to operate independently and the ability of governments to bend them to political will.^6^ Of note, across countries, scientists themselves are generally highly trusted and the agreement among people on scientific evidence is relatively large despite differences in personal beliefs.^7^ The problem lies instead with institutions and their governance.^6^

Strengthening the complex processes that support evidence production and establishing legal frameworks that guarantee institutional independence of evidence producers are likely among the most effective and sustainable responses to current attacks on public health science. Adequate resources, organizational structures, and a trained workforce are also needed.^4^ We need further to foster a respectful relationship between evidence producers and decision-makers to make possible the evidence-based policymaking dialogue. Scientific institutions led by experts in population health and clinical sciences—if protected from political interference—can build trust and engage constructively with stakeholders, and eventually “speak truth to the power” to help adopt policies informed by high-quality evidence.^2^ Ultimately, making possible the implementation of *Evidence in All Policies* will prevent a reversal of progress in population health and patients’ outcomes.
